# Global cocaine intoxication research trends during 1975–2015: a bibliometric analysis of Web of Science publications

**DOI:** 10.1186/s13011-017-0090-9

**Published:** 2017-02-02

**Authors:** Sa’ed H. Zyoud, W. Stephen Waring, Samah W. Al-Jabi, Waleed M. Sweileh

**Affiliations:** 1 Poison Control and Drug Information Center (PCDIC), College of Medicine and Health Sciences, An-Najah National University, Nablus, 44839 Palestine; 2 Department of Clinical and Community Pharmacy, College of Medicine and Health Sciences, An-Najah National University, Nablus, 44839 Palestine; 3 Acute Medical Unit, York Teaching Hospitals NHS Foundation Trust, Wigginton Road, York, YO31 8HE UK; 4 Department of Pharmacology and Toxicology, College of Medicine and Health Sciences, An-Najah National University, Nablus, 44839 Palestine

**Keywords:** Cocaine, Intoxication, Bibliometric, Web of Science

## Abstract

**Background:**

Cocaine is subject to recreational abuse as a stimulant and psychoactive agent, which poses a major worldwide health problem. The aim of the present study was to perform a bibliometric analysis of publication related to cocaine intoxication an insight of the research trends at a global level to enable recommendations for future research strategies in this field.

**Methods:**

Publications about cocaine intoxication were retrieved from the Web of Science (WoS) Core Collection database on December 28, 2016, and analysed regarding the following bibliometric indicators: research trends, document types, languages, countries/territories with their *h*-index, collaboration patterns, journals with their impact factors (IF), and institutions.

**Results:**

In total, 2,902 scientific publications from 1975 to 2015 were retrieved from the WoS database. The annual number of publications related to cocaine toxicity increased slightly after 1990 and reached a peak of 148 in 1992, with an average of 103 publications per year. The USA outranked other countries/territories with 2,089 publications, of which 1,927 arose exclusively from the USA and 162 involved international collaborations. The *h*-index for all publications related to cocaine was 212, and the *h*-index for all publications related to cocaine intoxication was 99. Moreover, the USA had the highest *h*-index of 95, followed by Spain with *h*-index of 24, and Canada with *h*-index of 24. The main research topics were consistently reproductive toxicity, clinical management of acute cocaine exposure, laboratory methods for detection of exposure to cocaine, cocaine metabolism, and cocaine toxicity in animals.

**Conclusions:**

This is the first bibliometric approach to examining research related to cocaine toxicity and shows that research activity has become more global and extensive since 1990. The USA remains the leading country regarding published literature, the highest *h*-index, and greatest role in international collaborations.

**Electronic supplementary material:**

The online version of this article (doi:10.1186/s13011-017-0090-9) contains supplementary material, which is available to authorized users.

## Background

Cocaine is subject to recreational abuse as a stimulant and psychoactive agent, and it is commonly presented in its hydrochloride form as a white, water-soluble powder, and may be used orally, intravenously or by nasal insufflation. Relatively pure formulations that lack a hydrochloride moiety are presented in a crystalline form, so-called ‘freebase’ or ‘crack’ cocaine, which may be used by nasal insufflation, smoking, ingestion or intravenous injection. Pharmaceutical cocaine preparations are available in countries that permit its use for medicinal purposes, namely as a local anaesthetic agent or to assist in managing epistaxis [1, 2].

Peak circulating cocaine concentrations occur almost immediately after intravenous injection and within several minutes of smoking, and may be delayed for up to 1 h after nasal insufflation. Cocaine is rapidly eliminated, with a half-life is around one hour and reported duration of acute effects between 2 and 4 h [2]. People who ingest cocaine may often be considered in two categories: body “stuffers” and body “packers”. Body stuffers may ingest moderate quantities of cocaine, often loosely packaged and, for example, ingested impulsively to avoid detection. Body packers typically ingest very large quantities of cocaine contained in multiple well wrapped packages for the purposes of drug smuggling. Body stuffers and body packers are at risk of systemic cocaine toxicity, and there may be severe or fatal poisoning due to gastrointestinal absorption after disruption of packed cocaine wrapping [3–6].

Pharmacological mechanisms of cocaine include blockade of sodium and potassium channels within the central nervous system, excess sympathetic autonomic outflow, and direct alpha adrenoceptor-mediated vasoconstriction of peripheral blood vessels [7]. Cocaine increases the risk of thrombotic and non-thrombotic acute coronary syndrome, stroke and arterial dissection and regular users have more advanced atherosclerosis than age-matched controls; cardiotoxicity is enhanced in users that co-ingest ethanol due to formation of cocaethylene [8]. These adverse effects include tachycardia, hypertension, chest pain, myocardial infarction, aortic and coronary artery dissection, QT prolongation due to potassium channel blockade, and arrhythmia including ventricular fibrillation [9, 10]. Other effects include sweating, fever, rhabdomyolysis, delirium, seizures, intracranial haemorrhage, and serotonin syndrome.

Powders and other chemicals are often added to increase bulk, including lidocaine, benzocaine, levamisole, baking flour, talc and washing powder, and microbial contaminants may also be present [11]. The observed effects may be caused by cocaine directly, or arise as an adverse effect of cutting agents or other contaminants. For example, agranulocytosis has been attributed to the presence of levamisole [12], and methaemoglobinaemia has been caused by local anaesthetic agents [13].

Clinical management of cocaine intoxication is supportive, including administration of benzodiazepines and high doses may be required to reduce agitation, treat seizures, and to allow control of tachycardia and high blood pressure. Fluid and electrolyte imbalance should be corrected and serial electrocardiographs and cardiac monitoring to assess for underlying myocardial ischaemia or dysrhythmia. Standard treatment for suspected cardiac ischaemia or myocardial infarction should be considered, namely antiplatelet agents, calcium channel blockers, nitrates, anticoagulants, and coronary arteriography [14, 15]. Intralipid may be considered for severe, life-threatening cardiac arrhythmia although too few data exist to fully understand its potential role in management of cocaine toxicity [16]. There has been controversy regarding the use of lidocaine due to its sodium channel blocking effects that might be expected to worsen cocaine cardiotoxicity; however, lidocaine may displace cocaine from cardiac sodium channels and reduce arrhythmia risk [17]. Beta-blockers are generally avoided as first line therapy because these will allow unopposed alpha adrenoceptor-mediated vasoconstriction, and should normally be used with caution after an alpha adrenoceptor blocker has been introduced [18].

At a global level, recreational cocaine use is at historically high levels [19–21]. Emerging trends demonstrate that cocaine use is having societal and health consequences. Bibliometric analysis is an efficient tool for examining trends in different scientific fields [22–26], and defined as the use of statistics and quantitative analysis for research output in the evaluation of research performance. Bibliometric network analysis allows analysis of research collaborations between countries, authors, and institutions [27–31].

Recently, bibliometric techniques have been used to explore trends in research related to various scientific disciplines; such as lab-on-a-chip research [32], nanotechnology research [33], public health research [34], organic farming research [35], pluripotent stem cell research [36], particulate matter and atherosclerosis research [37], and Helicobacter pylori research [38]. Earlier research has shown that the same methods may be applied to clinical toxicology themes such as intravenous lipid emulsion as an antidote [39], methanol poisoning [40], and calcium channel blockers poisoning [41]. To our knowledge, there has been no bibliometric study of research related to cocaine intoxication. The present study sought to apply established bibliometric techniques to the field of cocaine toxicity, to allow the overall research trends to be examined from a global perspective, and to help build recommendation for future research opportunities.

The study was designed to address the following questions: 1. What is the intellectual structure of the field of research that deals with cocaine intoxication? 2. What are the domains or subject clusters that are identified in this field, according to the terms used in publication titles and abstracts? 3. What has been the evolution of this field of research over time? 4. What are the main research topics related to cocaine toxicity, and connections between them? 5. What are the networks of researchers identified in the field, according to a co-authorship analysis? 6. What are the institutional networks in this field? 7. What are the main prolific journals, institutions, countries in this field? and, 8. Which publications have the highest impact on this field?

## Methods

Data about cocaine intoxication were retrieved from the Web of Science (WoS) Core Collection Database on December 28, 2016. This database is considered one of the most complete and reliable databases for bibliometric analyses, and covers over 12,000 of the highest impact, quality scientific international journals [42–46]. To identify research related to cocaine intoxication, we took the following steps in conducting this study:

- **Step 1:** Publications with “cocaine” as keywords in the title were downloaded. To achieve better accuracy in the results, the search was restricted to the Title field in the WoS database over all the previous year’s up to December 31, 2015 because if expanded to other search fields such as Abstract or Keywords, many publications obtained were not relevant to cocaine (i.e false-positive data). We applied a title-only search instead of a topic search (title, abstract, and keyword) accepting a small loss of sensitivity but significantly increasing specificity [35, 47]. Year 2016 was excluded as this year still open for new issues. Furthermore, data proposed to be incomplete due to reasons such as the time-lag between publications and indexing in WoS database. In this step it was promising to get all publications in the field of cocaine intoxication that were published in the period comprised between 1975 and 2015.
- **Step 2:** We limited our retrieved publications in the field of cocaine intoxication to all those indexed under the research category ‘Toxicology” in WoS database.
- **Step 3:** To include all the documents about cocaine intoxication that are published in journals or conference proceedings indexed in other subject categories, we used the following search strategy: term cocaine in the title; using the truncated terms *toxic*OR poison* OR overdos* as a search phrases to search topic in the WoS database over all the earlier years up to December 31, 2015. The search equation used produces publications that are relevant by truncating some terms, such as “poison*”, which leads to the recovery of publications on poison, poisoning, or poisonous. Furthermore, in this step, we excluded documents published in the category “Toxicology”.
- **Step 4:** In this step, search equations from step 1, 2 and 3 were combined in one search query and the results were analyzed and presented. Search query used for data extraction from WoS looked like this: (TI = (cocaine) AND TS = (*toxic* OR poison* OR overdos*)) OR (TI = (cocaine) AND SU = (Toxicology)); (See Additional file 1).
- **Step 5:** The retrieved publications were analysed regarding the following bibliometric indicators as done in previous bibliometric studies [27–31]: research trends, document types, languages, countries/territories with their *h*-index, collaboration patterns, journals with their impact factors (IF), and institutions. Our study relied on the connection between countries, topics, authors, and institutions using visualizations and clustering algorithms to locate the main groups among them by VOSviewer software [27, 48–50]. The VOSviewer v.1.6.5 was used for viewing and constructing the desired bibliometric maps [48]. VOSviewer was employed to illustrate the co-occurrence network of high-frequency terms related to cocaine toxicity to detect how research topics related to cocaine changed and progressed through time. The timespan of 1975–2015 was selected, and it was split into three periods: 1975–1995; 1996–2005, and 2006–2015. The size of circles in VOSviewer maps represents the number of publications related to certain term, and the distance between two terms gives an implication of the number of co-occurrences of the terms. Furthermore, terms close to each other or having certain color are more probable dealing with the same topic.

### Statistical analysis

All the retrieved results were imported into Excel 2007 for further analysis, and data presented as frequencies and percentages of publications. The ten most productive countries and journals in the field of cocaine toxicity were identified. The journal IF was obtained from the Journal Citation Report (JCR) Science Edition 2015. The *h*-index was calculated as the number of publications (n) that have achieved at least n citations. Publications originating from England, Northern Ireland, Scotland, and Wales were merged as being from the United Kingdom (UK). Pearson correlation test was used to examine the correlations between all cocaine publication productivity and that related to specifically to cocaine toxicity. A significance level of *P* < 0.05 was considered to be statistically significant. SPSS ® version 16 was used to perform the statistical analysis.

## Results

**Fig. 1:**
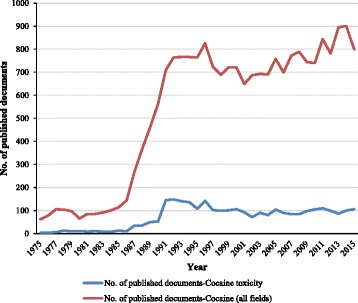
Evolution of scientific research in the field of cocaine toxicity

From 1975 to 2015, there were 21,683 publications on cocaine, including 2,902 scientific publications related to cocaine intoxication (See Additional file 1). Out of the 2,902 publications in the field of cocaine toxicity that were analysed in this study, 2,823 (97.3%) were published in English, followed by Spanish (36; 1.2%), French (29; 1.0%) and German (12; 0.4%). Original articles (2,205) were the most frequent publication type (76.0%), followed by meeting abstracts (323; 11.1%), proceedings papers (145, 5.0%), reviews (142; 4.9%), and letters (108; 3.7%). Annual publications on cocaine toxicity are summarised in Fig. 1. The annual number of publications related to cocaine toxicity increased slightly after 1990 and reached a peak of 148 publications in 1992, and then the total output has fluctuated with an average of 103 publications per year. Publication of articles related to cocaine in all fields has increased considerably after 1986 with a peak of 825 in 1996, and a subsequent average of 746 publications per year. There was a strong correlation between publication productivity related to cocaine in all fields and productivity related to cocaine toxicity (*r* = 0.929; *p*-value < 0.001).

Table 1 shows the leading countries/territories, ranked by number of publications. The USA outranked other countries/territories with 2,089 publications, of which 1,927 were exclusively produced in the USA and 162 were international collaborations. Spain published the second highest number of total publications with 145 publications, followed by Italy with 100 publications, Canada with 92 publications, and France with 90 publications. The *h*-index for all publications related to cocaine was 212, and the *h*-index for all publications related to cocaine intoxication was 99. Moreover, the USA had the highest *h*-index of 95, followed by Spain with *h*-index of 24, and Canada with *h*-index of 24. The highest average number of citations was for publications arising from the UK (32 citations), followed by the USA (25 citations), and Canada (25 citations).

**Table 1:** Top ten most productive countries in the field of cocaine toxicity

SCR	Country	Number of documents (%)	Average citations per document	*h*-index	No. of collaborative countries	No. of publications from collaboration
1^st^	USA	2089 (71.99)	25.15	95	38	162
2^nd^	Spain	145 (5.00)	13	24	14	30
3^rd^	Italy	100 (3.45)	15.12	23	10	28
4^th^	Canada	92 (3.17)	24.83	24	9	31
5^th^	France	90 (3.10)	14.31	21	13	30
6^th^	UK	80 (2.76)	32.33	23	13	32
7^th^	Brazil	55 (1.90)	8.32	12	8	16
8^th^	Germany	50 (1.72)	14.34	18	10	17
9^th^	Japan	42 (1.45)	13.19	14	1	9
10^th^	Switzerland	31 (1.07)	20.19	14	6	9

**Fig. 2:**
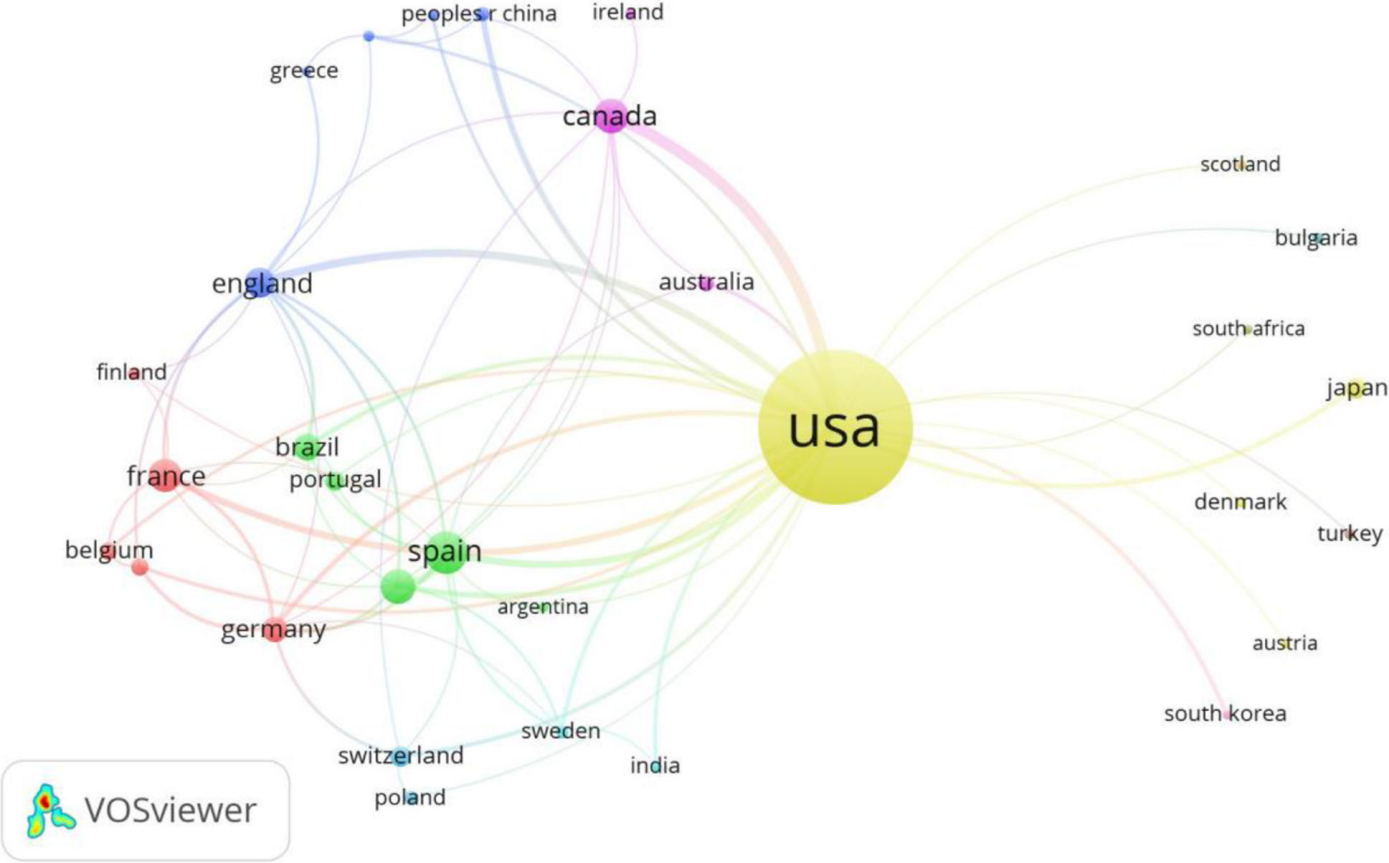
Network visualization map of country co-authorships. Of the 60 countries, 32 had at least five publications; the largest set of connected countries consists of 31 countries in 12 clusters

**Fig. 3:**
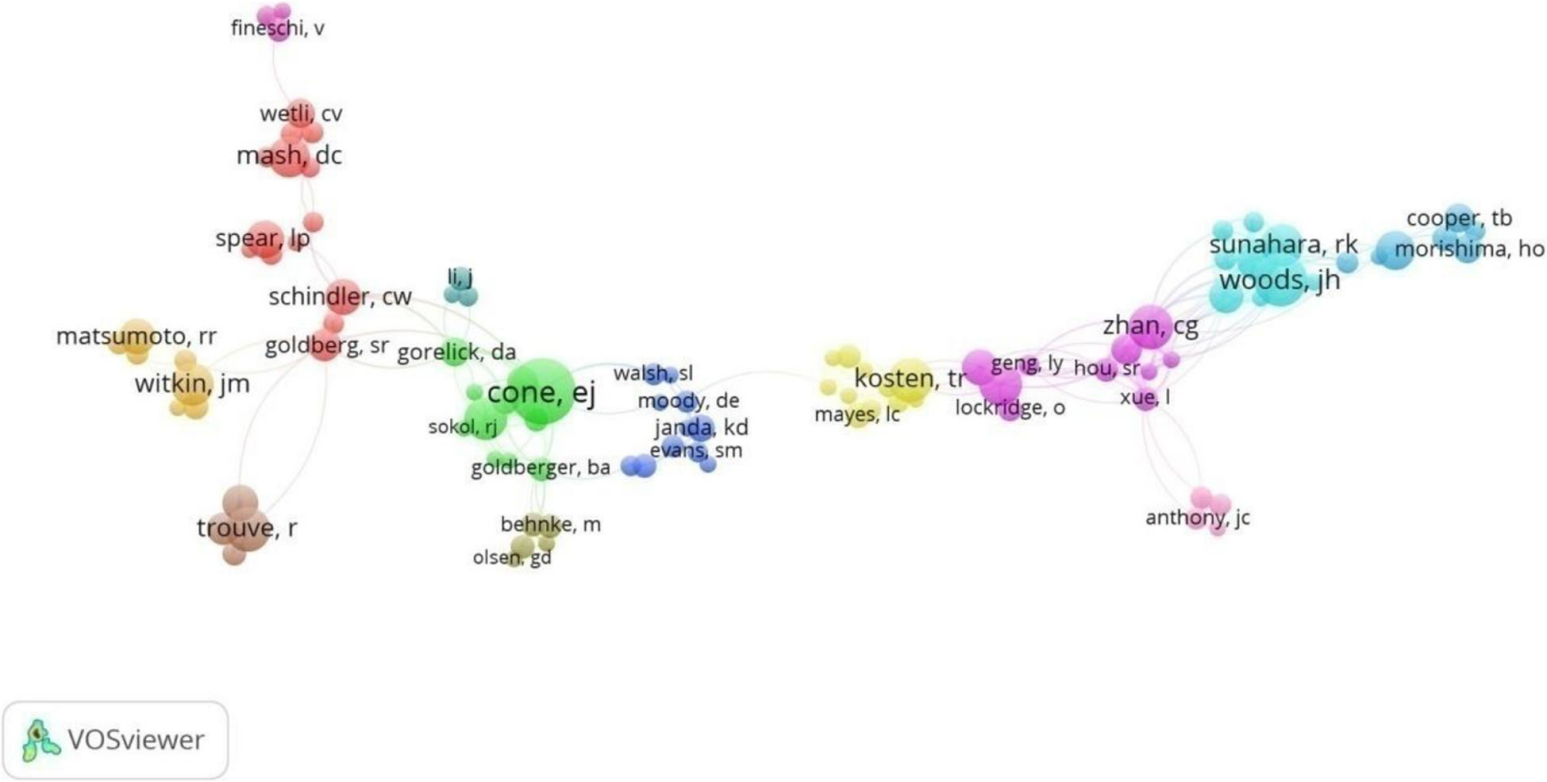
Network visualization map of the authors, 1975–2015. Of the 7,820 authors, 313 had at least five publications; the largest set of connected authors consists of 99 authors in 13 clusters

Figure 2 illustrates the collaboration network of countries publishing more than five documents. The size of circles represents the number of publications of the country and the thickness of lines signifies the size of collaboration. The USA had the most collaboration with other worldwide countries. A co-authorship map demonstrated that the top active authors in the field of cocaine intoxication were present in 13 different clusters (Fig. 3).

**Fig. 4:**
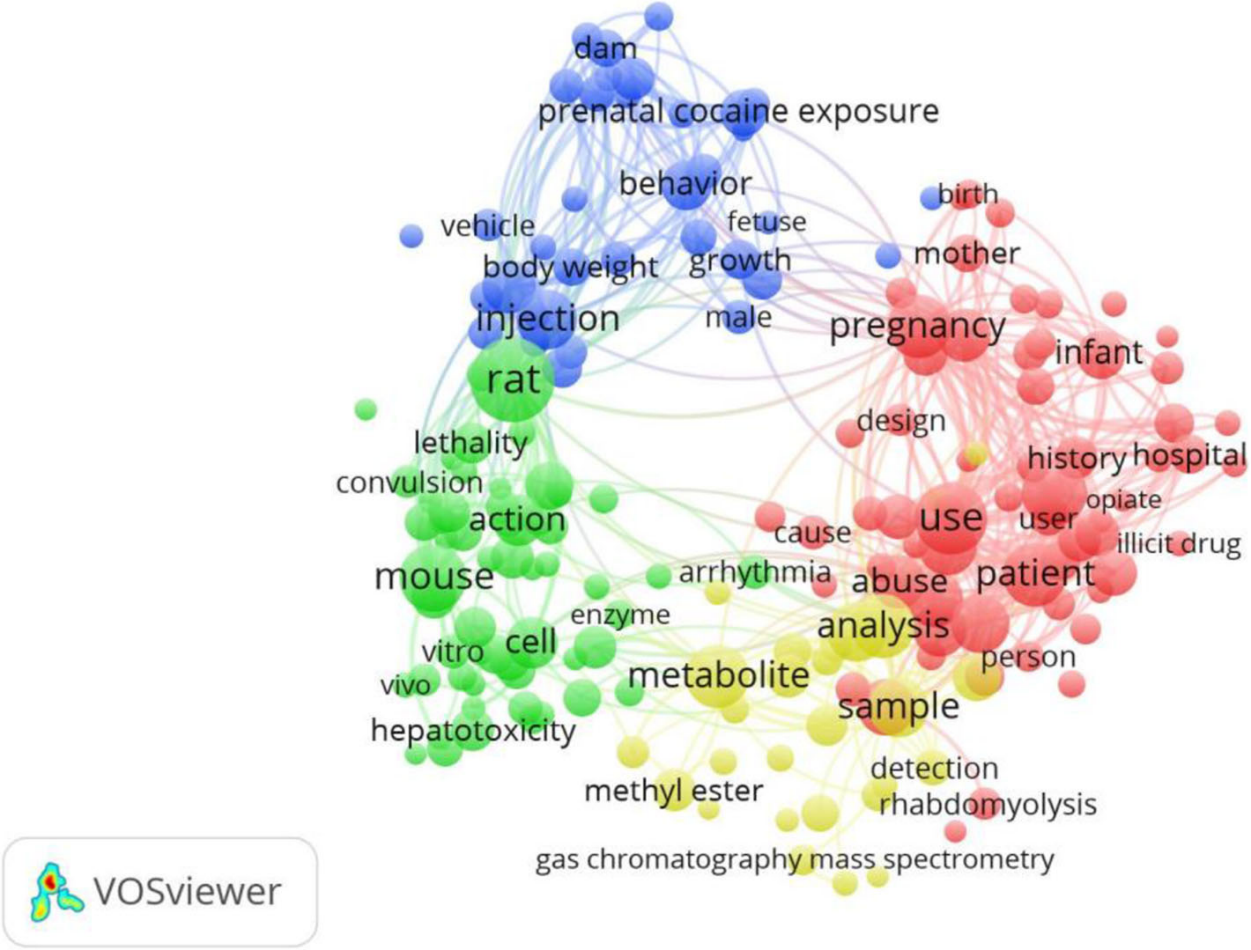
High-frequency terms in the titles and abstracts of cocaine toxicity publications during 1975–1995 with research topics indicated. Of the 11,752 terms, 303 terms occurred at least ten times. For each of the 303 terms, a relevance score was calculated and used to select the 60% most relevant terms. The largest set of connected terms consists of 182 terms in four clusters. (Number of publications related to cocaine intoxication = 954)

**Fig. 5:**
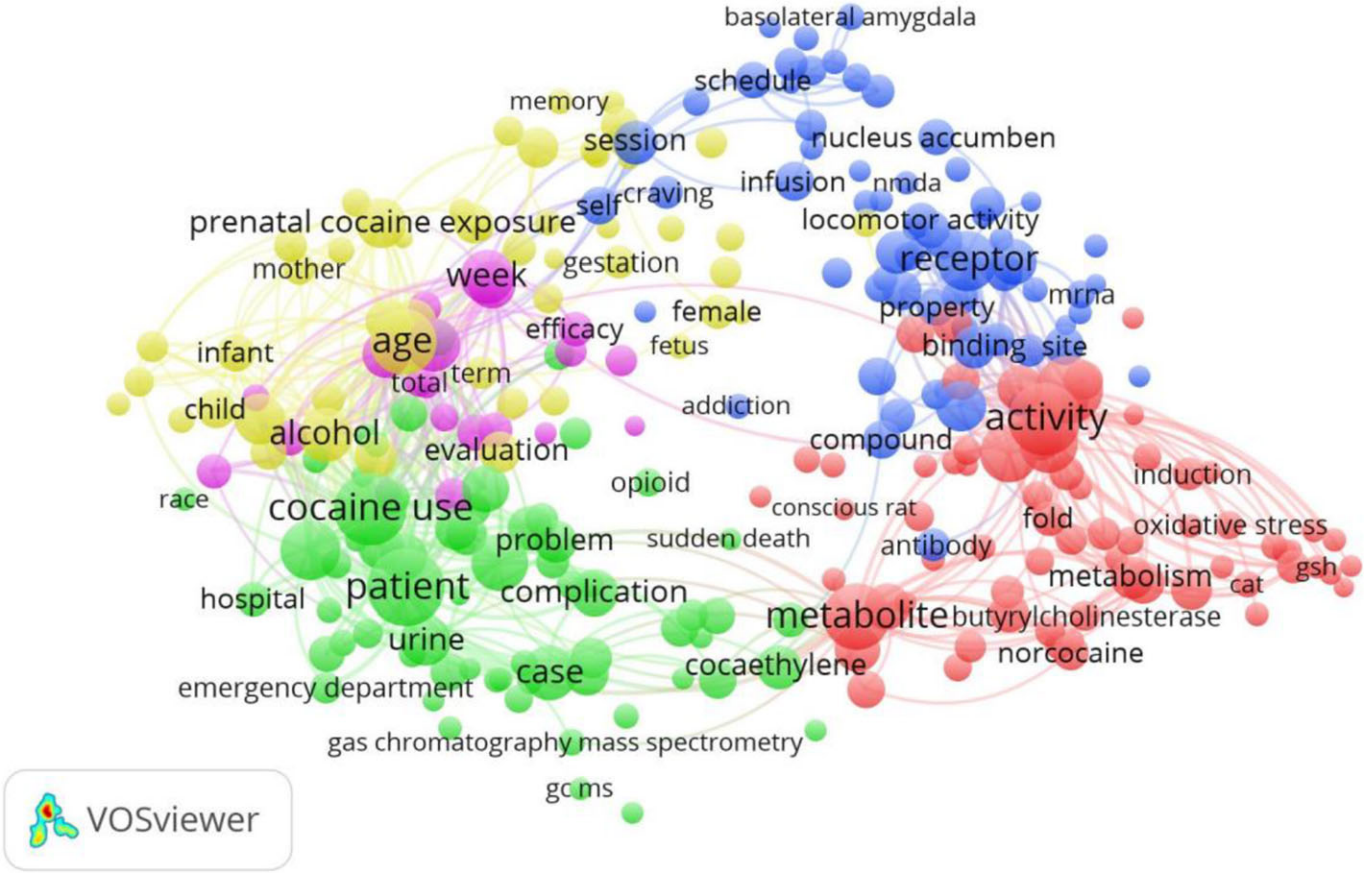
High-frequency terms in the titles and abstracts of cocaine toxicity publications during 1996–2005 with research topics indicated. Of the 17,767 terms, 451 terms were used at least ten times. For each of the 451 terms, a relevance score was calculated, and used to select the 60% most relevant terms. The largest set of connected terms consists of 271 terms in five clusters. (Number of publications related to cocaine intoxication = 987)

**Fig. 6:**
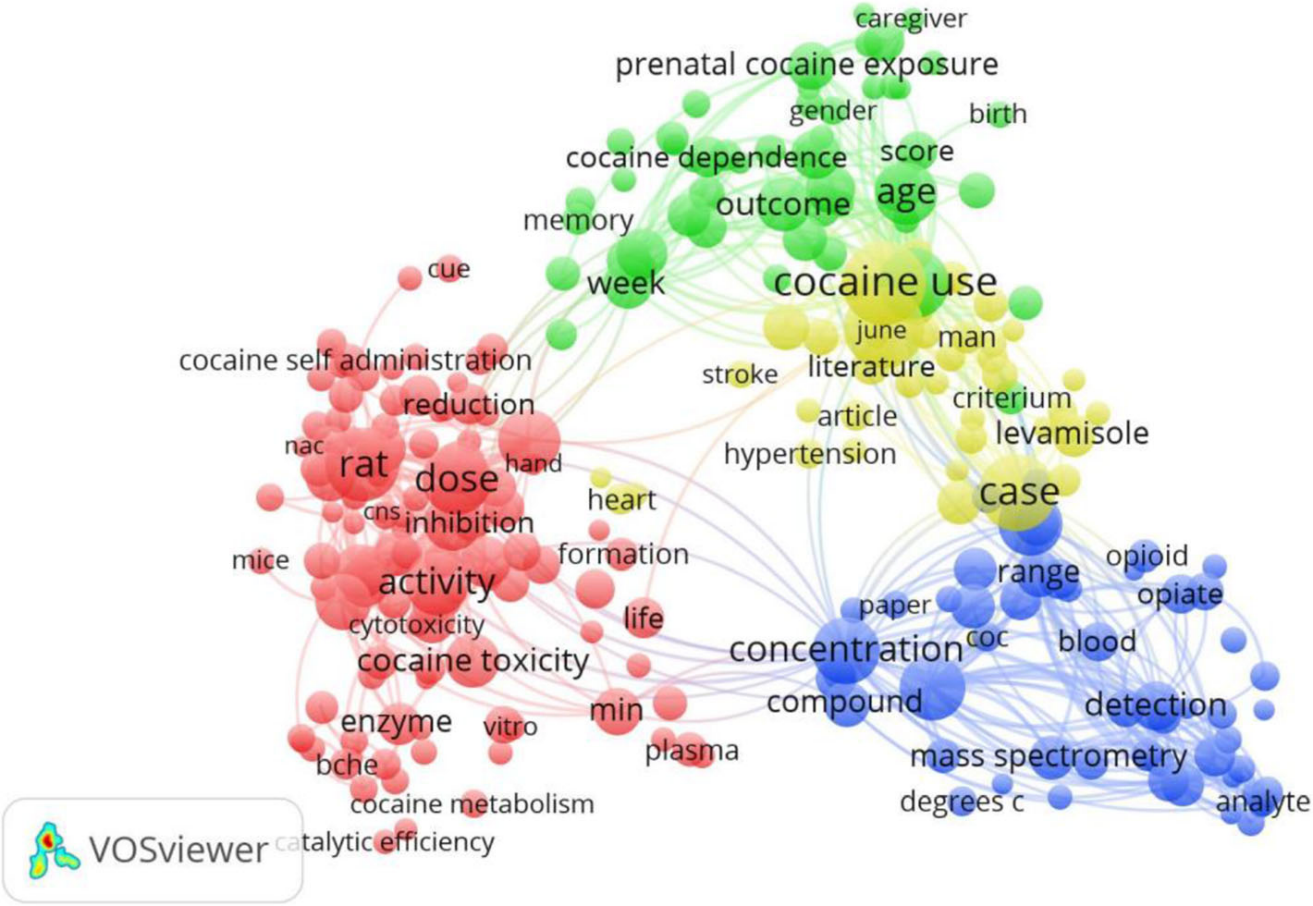
High-frequency terms in the titles and abstracts of cocaine toxicity publications during 2006–2015 with research topics indicated. Of the 16,914 terms, 440 terms were used at least ten times. For each of the 440 terms, a relevance score was calculated, and used to select the 60% most relevant terms. The largest set of connected terms consists of 264 terms in four clusters. (Number of publications related to cocaine intoxication = 961)

**Fig. 7:**
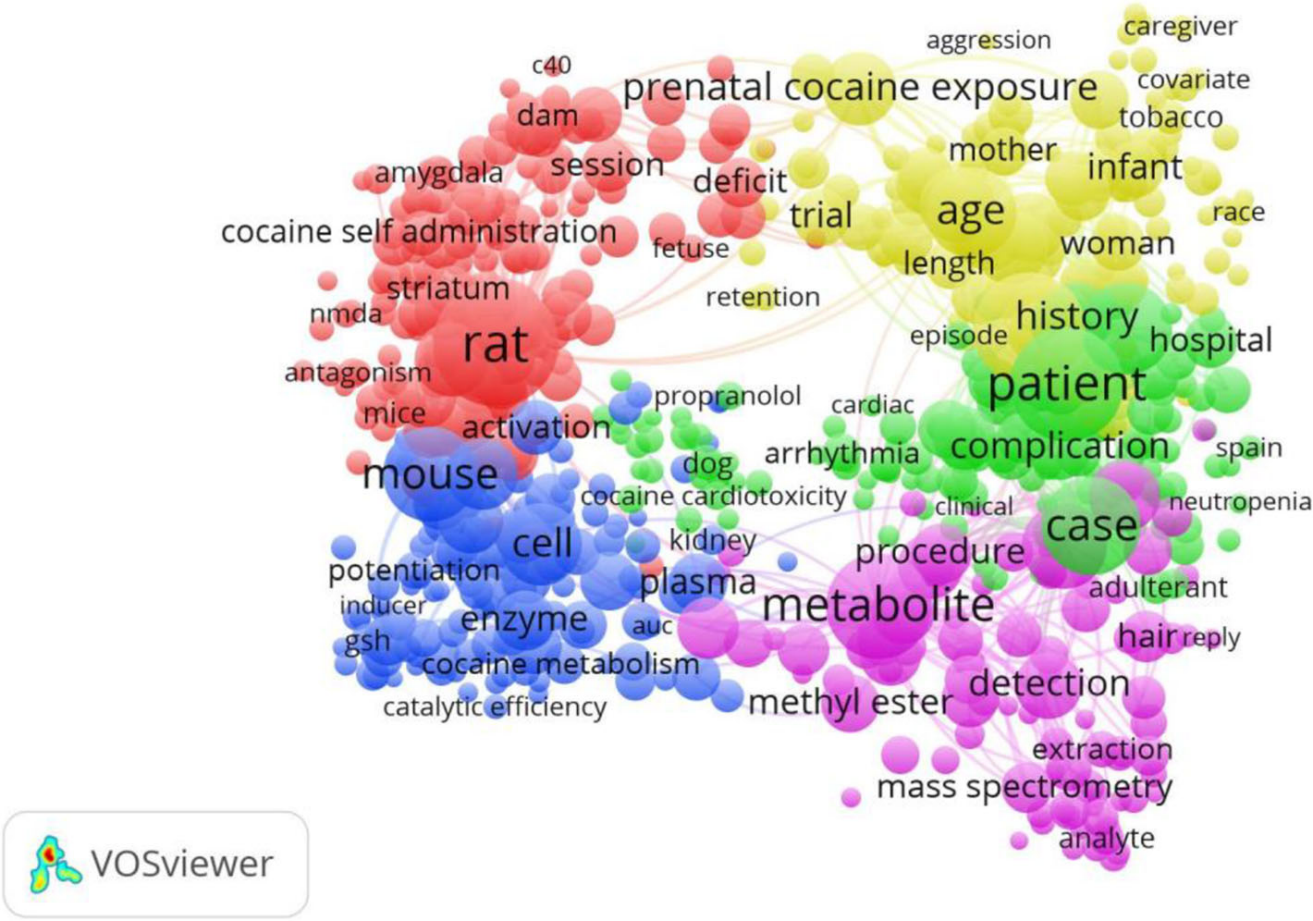
High-frequency terms in the titles and abstracts of cocaine toxicity publications during 1975–2015 with research topics indicated. Of the 38,273 terms, 1,135 terms occurred at least ten times. For each of the 1,135 terms, a relevance score was calculated, and used to select the 60% most relevant terms. The largest set of connected terms consists of 681 terms in five clusters. (Number of publications related to cocaine intoxication = 2,902)

The ten most productive of journals/periodicals in the field of cocaine toxicity are listed in Table 2. *Neurotoxicology and Teratology* published the most cocaine articles (274; 9.4%), followed by *Journal of Analytical Toxicology* (215; 7.4%), *Clinical Toxicology* (79; 2.7%), and *Journal of Pharmacology and Experimental Therapeutics* (64; 2.2%). The top ten most productive journals accounted for 33.5% of the total publications. The highest IF was associated with *Annals of Emergency Medicine* (IF = 5.008). Figures 4, 5, and 6 illustrate the co-occurrence networks for high-frequency terms related to cocaine toxicity in the titles and abstracts of the publications between 1975–1995, 1996–2005, and 2006–2015, respectively. Figure 7 shows the co-occurrence network of high-frequency terms in the title or/and abstract of retrieved publications related to cocaine toxicity during 1975–2015 which reflect most frequently encountered topics in this field. The five most used topics in cocaine toxicity are represented by five coloured clusters: blue, yellow, green, purple and red colors. Cluster number 1 (yellow color) included terms related to reproductive toxicity topic such as “prenatal cocaine exposure”, “infant”, or “mother”; Cluster number 2 (green color) included terms related to cocaine exposure and clinical management topic such as “patient”, “case”, or “hospital”; Cluster number 3 (purple color) included terms related to laboratory methods for detection of exposure to cocaine topic such as “mass spectrometry”, “metabolite”, or “detection”; Cluster number 4 (blue color) included terms related to cocaine metabolism topic such as “enzyme”, “inducer”, or “metabolism”; and Cluster number 5 (red color) included terms related to cocaine toxicity in animal models topic such as “rat”, “mice”. Additional file 2: Figure S1–S4 shows the density maps for co-occurrence of terms used in the title and abstract of retrieved publications across different time periods, from low density (blue) to high density (red).

**Table 2:** Ten most active journals in the field of cocaine toxicity

SCR	Journal/Periodical	Number of documents (%)	IF^a^
1^st^	Neurotoxicology and Teratology	274 (9.44)	2.488
2^nd^	Journal of Analytical Toxicology	215 (7.41)	2.322
3^rd^	Clinical Toxicology	79 (2.72)	2.886
4^th^	Journal of Pharmacology and Experimental Therapeutics	64 (2.21)	3.760
5^th^	Drug and Alcohol Dependence	60 (2.07)	3.349
6^th^	Annals of Emergency Medicine	52 (1.79)	5.008
6^th^	Journal of Forensic Sciences	52 (1.79)	1.322
8^th^	Toxicology Letters	50 (1.72)	3.522
9^th^	Pharmacology Biochemistry and Behavior	48 (1.65)	2.537
10^th^	Life Sciences	39 (1.34)	2.685
10^th^	Psychopharmacology	39 (1.34)	3.540

*SCR* Standard competition ranking, *IF* Impact factor

^a^The impact factor was reported according to journal citation reports (JCR) 2015

**Fig. 8:**
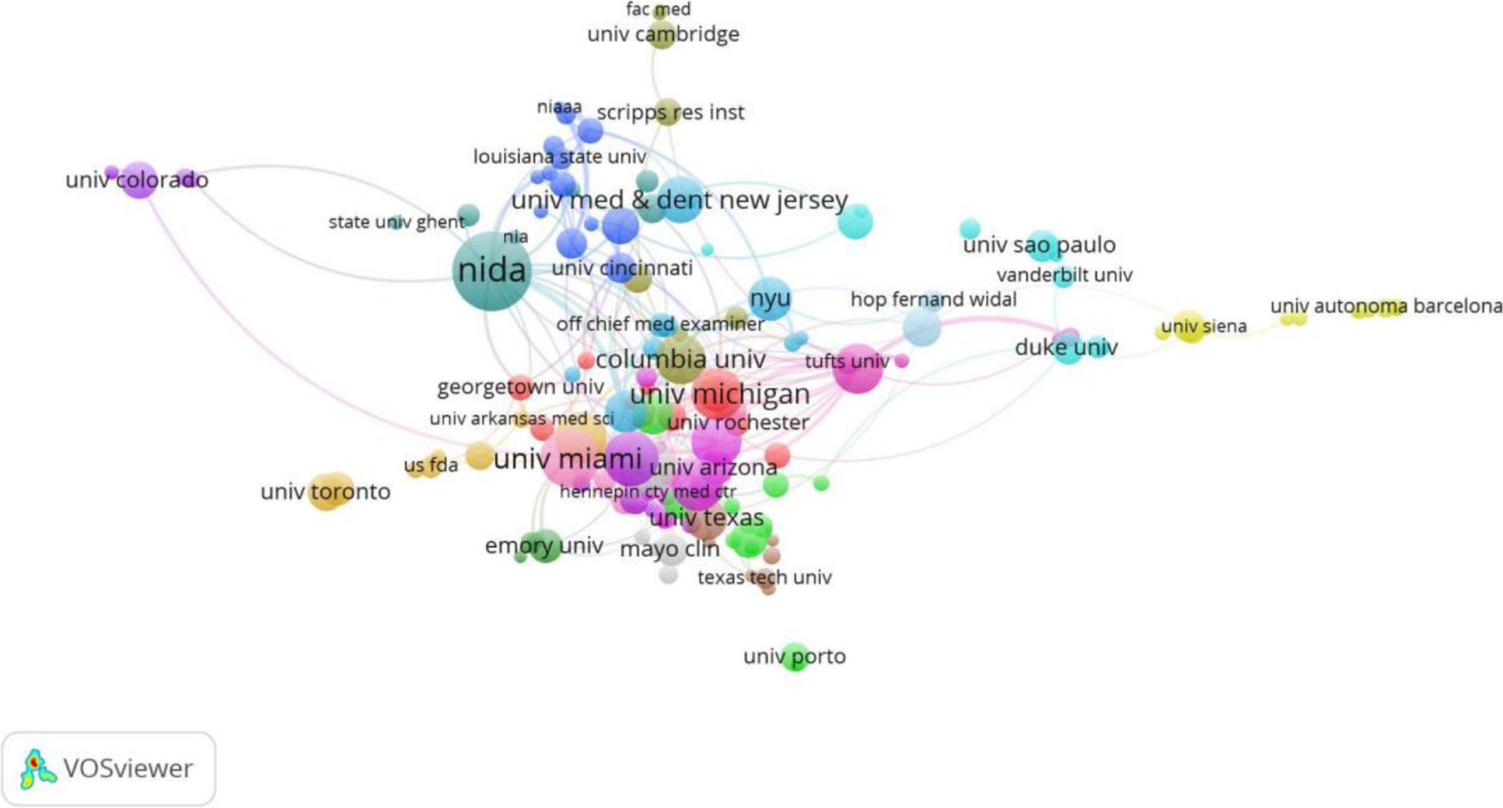
Institutional collaboration networks in cocaine toxicity during 1975–2015. Of the 1,533 institutes, 171 had at least five relevant publications. The largest set of connected institutes consists of 155 institutes in 18 clusters

The three most cited publications in cocaine toxicity are shown for each of the top ten productive countries (Table 3) [51–80]. From 1975 to 2015, the most frequently cited article was published in *Proceedings of the National Academy of Sciences* by Ramamoorthy et al [55] in 1993 and had been cited 657 times. Table 4 lists the top three most productive institutions from or collaborating with the top ten most productive countries in the field of cocaine toxicity. Leading was the *National Institute on Drug Abuse (NIDA)* with 115 publications, followed by *University of Miami* with 65 publications. Figure 8 demonstrates the collaboration network of top-155 institutes publishing more than five documents during 1975–2015. The size of circles represents the number of publications of the institute and the thickness of lines signifies the size of collaboration. As shown in Fig. 8, *National Institute on Drug Abuse (NIDA)*, *University of Miami,* and *Tufts University* have the most collaboration with other USA or worldwide institutes.

**Table 3:** Top three cited publications in the field of cocaine toxicity for the top 10 most productive countries

SCR	Authors	Title	Year of publication	Source title	IF^a^	Cited by	Average citations per year
USA							
1^st^	Ramamoorthy et al [55]	Antidepressant- and cocaine-sensitive human serotonin transporter: molecular cloning, expression, and chromosomal localization	1993	*Proceedings of the National Academy of Sciences*	9.423	657	27.38
2^nd^	Grant et al. [52]	Activation of memory circuits during cue-elicited cocaine craving	1996	*Proceedings of the National Academy of Sciences*	9.423	636	30.29
3^rd^	McFarland et al. [74]	Limbic and motor circuitry underlying footshock-induced reinstatement of cocaine-seeking behavior	2004	*Journal of Neuroscience*	5.924	304	23.38
Spain							
1^st^	Farre et al. [53]	Alcohol and cocaine interactions in humans	1993	*Journal of Pharmacology and Experimental Therapeutics*	3.760	133	5.54
2^nd^	Farre et al. [59]	Cocaine and alcohol interactions in humans: neuroendocrine effects and cocaethylene metabolism	1997	*Journal of Pharmacology and Experimental Therapeutics*	3.760	69	3.45
3^rd^	Ortega-Carnicer et al. [51]	Aborted sudden death, transient Brugada pattern, and wide QRS dysrrhythmias after massive cocaine ingestion	2001	*Journal of Electrocardiology*	1.290	63	3.94
Italy							
1^st^	Tagliaro et al. [58]	Capillary electrophoresis for the investigation of illicit drugs in hair: determination of cocaine and morphine	1993	*Journal of Chromatography A*	3.926	71	2.96
2^nd^	Addis et al. [68]	Fetal effects of cocaine: an updated meta-analysis	2001	*Reproductive Toxicology*	2.850	65	4.06
3^rd^	Cervo et al. [77]	Protein kinases A and C are involved in the mechanisms underlying consolidation of cocaine place conditioning	1997	*Brain Research*	2.561	58	2.90
Canada							
1^st^	Bozarth and Wise [79]	Toxicity associated with long-term intravenous heroin and cocaine self-administration in the rat	1985	*JAMA: Journal of the American Medical Association*	37.684	169	5.28
2^nd^	Nanji and Filipenko [56]	Asystole and ventricular fibrillation associated with cocaine intoxication	1984	*Chest*	5.94	169	5.12
3^rd^	Tyndale et al. [75]	Neuronal cytochrome P450IID1 (debrisoquine/sparteine-type): potent inhibition of activity by (-)-cocaine and nucleotide sequence identity to human hepatic P450 gene CYP2D6	1991	*Molecular Pharmacology*	3.931	143	5.50
France							
1^st^	Lenoir et al. [73]	Intense Sweetness Surpasses Cocaine Reward	2007	*PLOS ONE*	3.057	162	16.20
2^nd^	Maurice et al. [78]	Sigma(1) (sigma(1)) receptor antagonists represent a new strategy against cocaine addiction and toxicity	2002	*Neuroscience & Biobehavioral Reviews*	8.580	103	6.87
3^rd^	Pellinen et al. [62]	Cocaine N-demethylation and the metabolism-related hepatotoxicity can be prevented by cytochrome P450 3A inhibitors	1994	*European Journal of Pharmacology*	2.730	75	3.26
UK							
1^st^	Ito et al. [64]	Differential control over cocaine-seeking behavior by nucleus accumbens core and shell	2004	*Nature Neuroscience*	16.724	269	
2^nd^	Whitelaw et al. [67]	Excitotoxic lesions of the basolateral amygdala impair the acquisition of cocaine-seeking behaviour under a second-order schedule of reinforcement	1996	*Psychopharmacology*	3.540	244	
3^rd^	Vorel et al. [66]	Dopamine D-3 receptor antagonism inhibits cocaine-seeking and cocaine-enhanced brain reward	2002	*Journal of Neuroscience*	5.924	200	
Brazil							
1^st^	Masur et al. [72]	Increased stimulatory effect by the combined administration of cocaine and alcohol in mice.	1989	*Alcohol*	2.440	36	1.29
2^nd^	Crouch et al. [54]	Analysis of cocaine and its metabolites from biological specimens using solid-phase extraction and positive ion chemical ionization mass spectrometry	1995	*Journal of Analytical Toxicology*	2.322	34	1.55
3^rd^	Lepsch et al. [61]	Cocaine induces cell death and activates the transcription nuclear factor kappa-b in pc12 cells	2009	*Molecular Brain*	3.745	30	3.75
Germany							
1^st^	Wilbert-Lampen et al. [60]	Cocaine increases the endothelial release of immunoreactiveendothelin and its concentrations in human plasma and urine - Reversal by coincubation with sigma-receptor antagonists	1998	*Circulation*	17.047	69	3.63
2^nd^	Maurer et al. [80]	Toxicokinetics of drugs of abuse: Current knowledge of the isoenzymes involved in the human metabolism of tetrahydrocannabinol, cocaine, heroin, morphine, and codeine	2006	*Therapeutic Drug Monitoring*	2.094	67	6.09
3^rd^	BrenzVerca et al. [65]	Distribution of alpha- and gamma-synucleins in the adult rat brain and their modification by high-dose cocaine treatment	2003	*European Journal of Neuroscience*	2.975	40	2.86
Japan							
1^st^	Nakahara et al. [69]	Hair analysis for drugs of abuse. V. The facility in incorporation of cocaine into hair over its major metabolites, benzoylecgonine and ecgonine methyl ester	1992	*Archives of Toxicology*	6.637	69	2.76
2^nd^	Aoki et al. [63]	Cocaine-induced liver injury in mice is mediated by nitric oxide and reactive oxygen species	1997	*European Journal of Pharmacology*	2.730	45	2.25
3^rd^	Nakahara and Kikura [70]	Hair analysis for drugs of abuse. VII. The incorporation rates of cocaine, benzoylecgonine and ecgonine methyl ester into rat hair and hydrolysis of cocaine in rat hair	1994	*Archives of Toxicology*	6.637	43	1.87
Switzerland							
1^st^	Boelsterli and Goldlin [57]	Biomechanisms of cocaine-induced hepatocyte injury mediated by the formation of reactive metabolites	1991	*Archives of Toxicology*	6.637	95	3.65
2^nd^	Boelsterli et al. [71]	Identification of cytochrome P-450IIB1 as a cocaine-bioactivating isoform in rat hepatic microsomes and in cultured rat hepatocytes.	1992	*Drug Metabolism & Disposition*	3.210	62	2.48
3^rd^	Boelsterli et al. [76]	Oxygen free radical production mediated by cocaine and its ethanol-derived metabolite, cocaethylene, in rat hepatocytes.	1993	*Hepatology*	11.711	54	2.25

*SCR* Standard competition ranking, *IF* Impact factor

^a^The impact factor was reported according to journal citation reports (JCR) 2015

**Table 4:** Top three most productive institutions from or collaborating with the top ten most productive countries in the field of cocaine toxicity

SCR	Institute	*n* (%)
USA (number of documents = 2,089)		
1^st^	*National Institute on Drug Abuse (NIDA)*	115 (5.51)
2^nd^	*University of Miami*	65 (3.11)
3^rd^	*Yale University*	55 (2.63)
Italy(number of documents = 100)		
1^st^	*UniversitàCattolica del Sacro Cuore*	12 (12.00)
2^nd^	*IstitutoSuperiore di Sanità*	9 (9.00)
3^rd^	*Sapienza – Università di Roma*	8 (8.00)
France(number of documents = 90)		
1^st^	*HôpitalFernand-Widal*	15 (16.67)
2^nd^	*Institut national de la santé et de la recherchemédicale-INSERM*	9 (10.00)
3^rd^	*Columbia University College of Physicians and Surgeons*	7 (7.78)
Brazil(number of documents = 55)		
1^st^	*University of São Paulo*	22 (40.00)
2^nd^	*Universidade Federal de Minas Gerais*	6 (10.91)
3^rd^	*Universidade Federal de São Paulo*	6 (10.91)
Japan(number of documents = 42)		
1^st^	*Kyoto University*	9 (21.43)
2^nd^	*Showa University*	6 (14.29)
3^rd^	*National Institute on Drug Abuse (NIDA)*	4 (9.52)
Spain (number of documents = 145)		
1^st^	*University of Santiago de Compostela*	15 (10.35)
2^nd^	*Autonomous University of Barcelona*	11 (7.59)
3^rd^	*University of Valencia*	9 (6.21)
Canada(number of documents = 92)		
1^st^	*University of Toronto*	28 (30.44)
2^nd^	*The Hospital for Sick Children*	24 (26.09)
3^rd^	*University of British Columbia*	8 (8.70)
UK(number of documents = 80)		
1^st^	*University of Cambridge*	18 (22.50)
2^nd^	*Guy’s and St Thomas’ NHS Foundation Trust*	6 (7.50)
3^rd^	*Guy’s Hospital*	4 (5.00)
Germany(number of documents = 50)		
1^st^	*Goethe University Frankfurt*	4 (8.00)
2^nd^	*Maastricht University*	4 (8.00)
3^rd^	*Universität Heidelberg*	4 (8.00)
Switzerland(number of documents = 31)		
1^st^	*University of Zurich*	12 (38.71)
2^nd^	*Swiss Federal Institute of Technology*	10 (32.26)
3^rd^	*Université de Fribourg*	2 (6.45)

*n* Number of documents (%), *SCR* Standard competition ranking

## Discussion

The number of publications in the field of cocaine intoxication has grown during the studied 40 years, and correlates with growth in publications in all fields related to cocaine. There was a steady growth rate until 1992, then total output has been fairly stable with some fluctuations between 1992 and 2015. The increased number of publications may be due to: 1. the number of frequent cocaine users has been increasing since 1980 [81], 2. greater awareness of concerns about life-threatening consequences of cocaine toxicity, particularly related to cardiac, cerebrovascular, and maternal morbidity and mortality [2, 82, 83], 3. increasing cocaine use at a global level [84], and, 4. new pharmacological concepts related to cocaine use, namely in its role as a local anaesthetic agent [81, 85].

The USA is the most productive country in research related to cocaine intoxication, which is similar to patterns identified for other clinical toxicology research, such as intravenous lipid emulsion as an antidote [39], paracetamol poisoning [86, 87], acetylcysteine as antidote [88], methanol poisoning [40], and calcium channel blockers poisoning [41]. Possible reasons include the comparatively large research budgets, and rapid economic growth [89]. In addition, it was reported that cocaine was the most commonly abused drug in parts of the USA [1, 19]. Another noticeable finding was that all the top cited publications in the field of cocaine intoxication originated from the USA. These results are consistent with data obtained by previous bibliometric studies that a few developed countries such as the USA generate the most frequently cited toxicology studies [39, 41, 87, 90]. This might be influenced by factors such as access to publications by scholars from the USA. There may be greater opportunities for USA researchers to access databases and attend international conferences and academic exchange programs, that contribute to higher citation rates [91]. A possible explanation is the generalised trend towards increasing publication numbers across a range of scientific fields within the USA. Furthermore, there are some indications that the USA researchers tend to cite publications from their own country [92].

The percentages of all publications appearing in the top journals were comparatively low, indicating a spread of publications allocated to generalised and specialised journals, and reflecting the broad range of research interests related to cocaine. This is similar to several other areas of toxicology research with a high level of multidisciplinary interest, including intravenous lipid emulsion as an antidote [39], methanol poisoning [40], and calcium channel blockers poisoning [41].

The most frequently cited and highly influential publication was related to a novel hypothesis of cocaine pharmacological action, namely “Antidepressant- and cocaine-sensitive human serotonin transporter: molecular cloning, expression, and chromosomal localization”. Understanding the citation patterns is important in evaluating an individual publication, and may also help understand how a certain topics or concepts are disseminated within the scientific community [93].

Bibliometric analysis has a limitations, including database variations, discipline variation, and bias towards English language [26, 94]. As with all previous bibliometric studies [94–96], our study is limited by use of search term “cocaine” to only the title search. Specially, any publications that used “cocaine” as a key word in the publication may have been missed in our analysis. It is widely known that the total number of publications from major databases such as Google Scholar, Scopus, PubMed, and WoS differs. Furthermore, there is an indisputable inclination that English is the language of science, and certain databases may omit publications in different languages.

## Conclusions

Research progress related to cocaine intoxication has been assessed for the first time based on a bibliometric approach. Research related to cocaine intoxication has become more global and extensive after 1990, and the USA is the leading country with the greatest number of publications and highest *h*-index. The main topics have consistently been reproductive toxicity, cocaine exposure and clinical management, laboratory methods for detection of exposure to cocaine, cocaine metabolism, and cocaine toxicity in animals. These findings may provide a valuable basis for identifying important topics for future research, and create opportunities for collaboration between research groups with complementary scientific interest in the field of cocaine toxicity.

## Additional files

Additional file 1: Methodology used to retrieve publications related to cocaine intoxication for analysis using Web of Science (WoS) Core Collection Database. (DOCX 17 kb)
Additional file 2: Density view of terms map based on the co-occurrence matrix of terms from text data in the title and abstract of retrieved publications related to cocaine toxicity by periods. **Figure S1.** Density view of terms map in Period I (1975–1995); colors show the density of relevance, sorting from blue (lowest density) to red (highest density). (number of publications related to cocaine intoxication = 954). **Figure S2.** Density view of terms map in Period II (1996–2005); colors show the density of relevance, sorting from blue (lowest density) to red (highest density). (Number of publications related to cocaine intoxication = 987). Figure S3. Density view of terms map in Period III (2006–2015); colors show the density of relevance, sorting from blue (lowest density) to red (highest density). (Number of publications related to cocaine intoxication = 961). **Figure S4.** Density view of terms map in Period 1975–1995; colors show the density of relevance, sorting from blue (lowest density) to red (highest density). (Number of publications related to cocaine intoxication = 2,902). (DOCX 794 kb)

## Abbreviations

IFs: Impact factors
JCR: Journal Citation Reports
SCR: Standard Competition Ranking
WoS: Web of Science
