# Supplementary material for: Global cocaine intoxication research trends during 1975–2015: a bibliometric analysis of Web of Science publications
Source: Subst Abuse Treat Prev Policy. 2017 Feb 2;12:6. doi: 10.1186/s13011-017-0090-9 (PMC5290655; doi:10.1186/s13011-017-0090-9)
Supplement: Additional file 1: — Methodology used to retrieve publications related to cocaine intoxication for analysis using Web of Science (WoS) Core Collection Database. (DOCX 17 kb) [file 13011_2017_90_MOESM1_ESM.docx]

**Additional file 1** Methodology used to retrieve publications related to cocaine intoxication for analysis using Web of Science (WoS) Core Collection Database

You searched for: (TI=(cocaine) AND TS=(*toxic* OR poison* OR overdos*)) OR (TI=(cocaine) AND SU=(Toxicology))

Timespan: 1975-2015

Number of publications = 2,902

You searched for: TITLE: (cocaine)

Timespan: 1975-2015

Number of publications= 21,683

You searched for: TOPIC: (cocaine)

Timespan: 1975-2015

Number of publications= 45,123
