# Supplementary material for: Global cocaine intoxication research trends during 1975–2015: a bibliometric analysis of Web of Science publications
Source: Subst Abuse Treat Prev Policy. 2017 Feb 2;12:6. doi: 10.1186/s13011-017-0090-9 (PMC5290655; doi:10.1186/s13011-017-0090-9)
Supplement: Additional file 2: — Density view of terms map based on the co-occurrence matrix of terms from text data in the title and abstract of retrieved publications related to cocaine toxicity by periods. Figure S1. Density view of terms map in Period I (1975–1995); colors show the density of relevance, sorting from blue (lowest density) to red (highest density). (number of publications related to cocaine intoxication = 954). Figure S2. Density view of terms map in Period II (1996–2005); colors show the density of relevance, sorting from blue (lowest density) to red (highest density). (Number of publications related to cocaine intoxication = 987). Figure S3. Density view of terms map in Period III (2006–2015); colors show the density of relevance, sorting from blue (lowest density) to red (highest density). (Number of publications related to cocaine intoxication = 961). Figure S4. Density view of terms map in Period 1975–1995; colors show the density of relevance, sorting from blue (lowest density) to red (highest density). (Number of publications related to cocaine intoxication = 2,902). (DOCX 794 kb) [file 13011_2017_90_MOESM2_ESM.docx]

**Additional file 2** (Figure S1, S2, S3, and S4) Density view of terms map based on the co-occurrence matrix of terms from text data in the title and abstract of retrieved publications related to cocaine toxicity by periods.


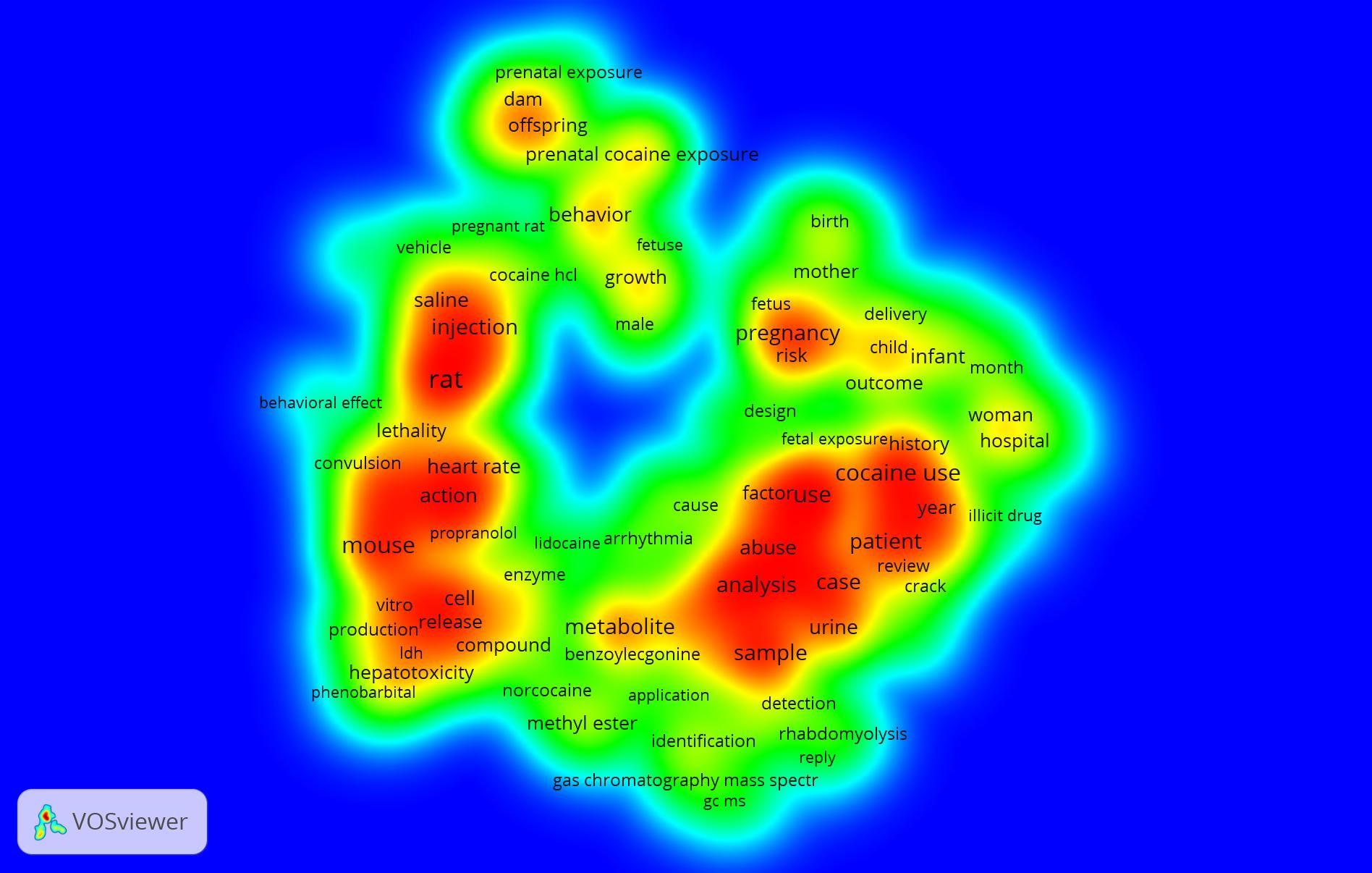


**Figure S1** Density view of terms map in Period I (1975-1995); colors show the density of relevance, sorting from blue (lowest density) to red (highest density). (number of publications related to cocaine intoxication = 954)

**
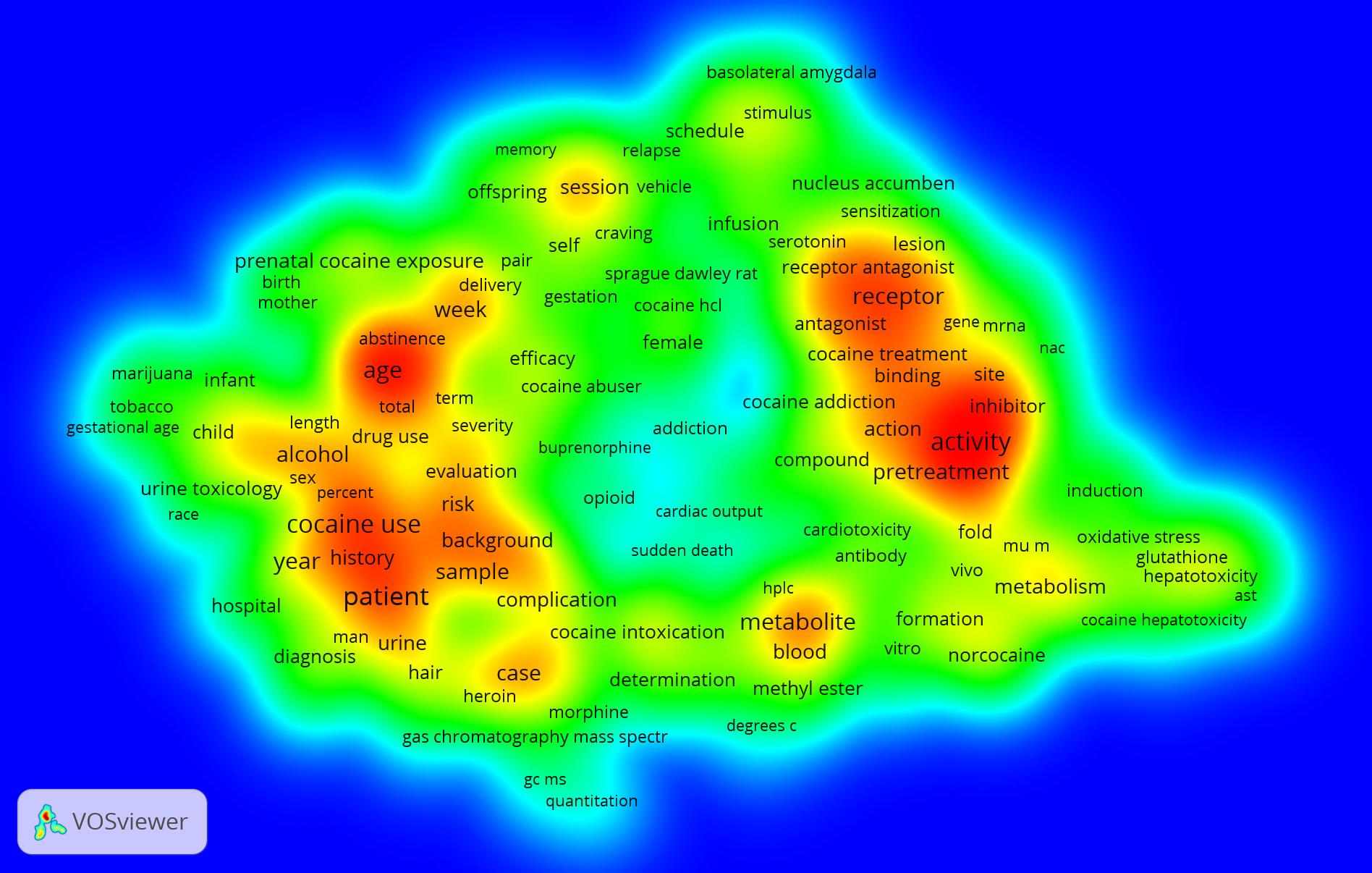
**

**Figure S2** Density view of terms map in Period II (1996-2005); colors show the density of relevance, sorting from blue (lowest density) to red (highest density). (Number of publications related to cocaine intoxication = 987)

**
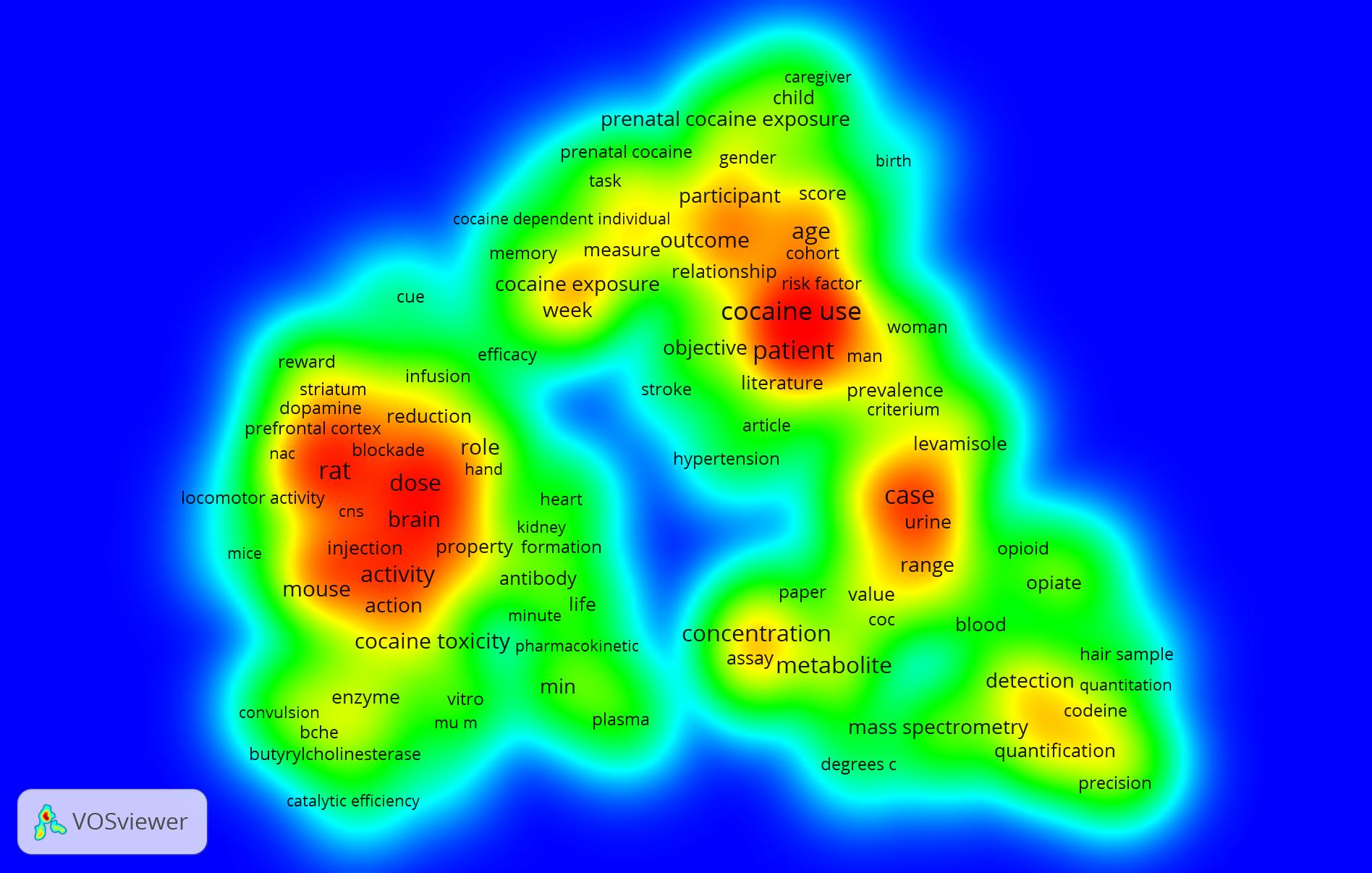
**

**Figure S3** Density view of terms map in Period III (2006-2015); colors show the density of relevance, sorting from blue (lowest density) to red (highest density). (Number of publications related to cocaine intoxication = 961)

**
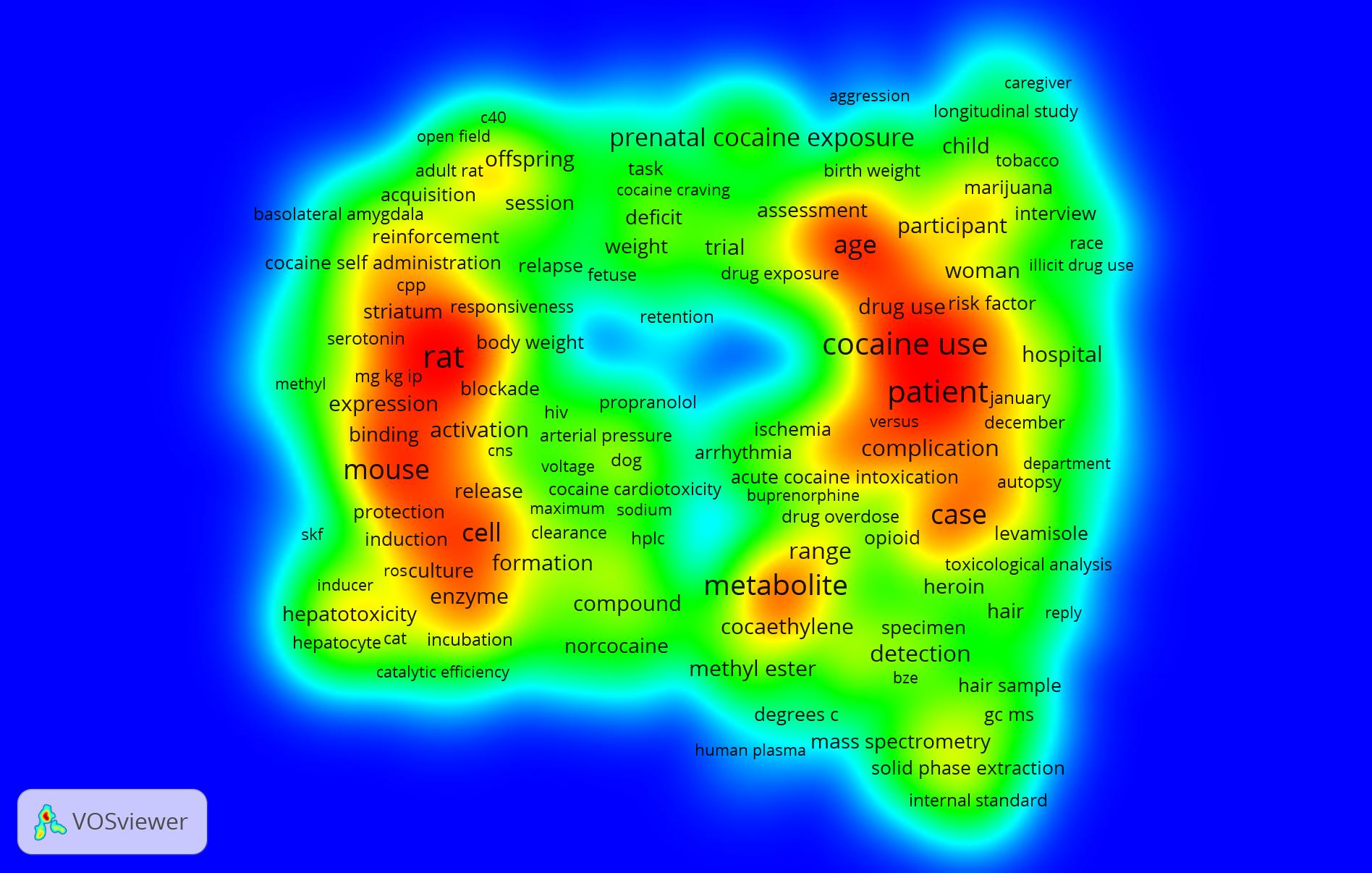
**

**Figure S4** Density view of terms map in Period 1975-1995; colors show the density of relevance, sorting from blue (lowest density) to red (highest density). (Number of publications related to cocaine intoxication = 2,902)
